# Driver Visual Attention Before and After Take-Over Requests During Automated Driving on Public Roads

**DOI:** 10.1177/00187208221093863

**Published:** 2022-06-16

**Authors:** Linda Pipkorn, Marco Dozza, Emma Tivesten

**Affiliations:** 11248 Chalmers University of Technology, Gothenburg, Sweden and Volvo Cars, Gothenburg, Sweden

**Keywords:** vehicle automation, visual attention, take-over request, human-automation interaction, automated driving, driver behavior

## Abstract

**Objective:**

This study aims to understand drivers’ visual attention before and after take-over requests during automated driving (AD), when the vehicle is fully responsible for the driving task on public roads.

**Background:**

Existing research on transitions of control from AD to manual driving has mainly focused on take-over times. Despite its relevance for vehicle safety, drivers’ visual attention has received little consideration.

**Method:**

Thirty participants took part in a Wizard of Oz study on public roads. Drivers’ visual attention was analyzed before and after four take-over requests. Visual attention during manual driving was also recorded to serve as a baseline for comparison.

**Results:**

During AD, the participants showed reduced visual attention to the forward road and increased duration of single off-road glances compared to manual driving. In response to take-over requests, the participants looked away from the forward road toward the instrument cluster. Levels of visual attention towards the forward road did not return to the levels observed during manual driving until after 15 s had passed.

**Conclusion:**

During AD, drivers may look toward non-driving related task items (e.g., mobile phone) instead of forward. Further, when a transition of control is required, drivers may take over control before they are aware of the driving environment or potential threat(s). Thus, it cannot be assumed that drivers are ready to respond to events shortly after the take-over request.

**Application:**

It is important to consider the effect of the design of take-over requests on drivers’ visual attention alongside take-over times.

## INTRODUCTION

Vehicles equipped with assisted driving systems (generally equivalent to SAE Level 2 driving automation) are available on public roads today (SAE International, 2018; Thatcham Research, 2019). These systems assist the drivers with parts of the driving task (e.g., steering/braking), but the driver is always the one responsible for safe driving. Future automated driving (AD) systems (generally equivalent to SAE Level 4), on the other hand, promise to handle the driving task to such an extent that drivers do not need to supervise the system (SAE International, 2018; Thatcham Research, 2019). Consequently, drivers can drive without their hands on the steering wheel, feet on the pedals, or eyes on the forward road—as long as AD is active. However, the drivers are expected to be receptive to automation-issued take-over requests (TORs) and to respond appropriately to these requests (SAE International, 2018; United Nations Economic Comission for Europe, 2021).

### Take-over Times and Visual Attention 

The expectation that drivers can respond appropriately to TORs by safely resuming manual control has been questioned by human factors researcher (Gold et al., 2013; Louw et al., 2015; Seppelt & Victor, 2016). To date, drivers’ responses to TORs have been explored mainly in driving simulators (Gold et al., 2013; Louw et al., 2015; McDonald et al., 2019), and to a lesser extent in more realistic settings such as real roads (Eriksson et al., 2017; Naujoks et al., 2019) or test tracks (Pipkorn et al., 2021, 2022). The focus of the current research is the time needed for drivers to deactivate AD by steering, braking, or pressing buttons (the take-over time; McDonald et al., 2019). Thus, how well drivers can respond to TORs has typically been evaluated through this time needed for their motor response, while drivers’ visual attention towards the forward roadway before and after TORs have received less consideration.

However, it is important to understand drivers’ visual attention, because safe manual driving (including after a transition of control from AD) requires drivers to look at the right place at the right time—usually the forward roadway, in the absence of intersections (Victor et al., 2005, 2015). While off-road glances longer than 2 s during manual driving have been associated with increased risk of crashing, shorter off-road glances can also lead to crashing if they coincide with sudden changes in the environment, such as a braking lead vehicle (Victor et al., 2015). Consequently, drivers cannot be expected to respond appropriately to unexpected events shortly after a TOR until their visual attention to the road is restored to the levels typical for manual driving.

###  Visual Attention During Assisted and Automated Driving

Previous research indicates that automating parts of the driving task may lead drivers to look less towards the forward roadway than they do during manual driving. For example, previous research on test tracks and in real traffic reports that percent road center (PRC: the percentage of time that a driver’s gaze is directed toward the forward road; Victor et al., 2005) is lower during assisted driving than during manual driving (Tivesten et al., 2015, 2019). In addition, drivers also tend to exhibit slightly longer off-road glance durations during assisted driving (see Gaspar & Carney, 2019; Morando et al., 2019) than in manual mode. Victor et al. (2018) reported off-road glances up to 40 s during assisted driving when no attention reminders were given, although drivers were still responsible for supervising the vehicle.

Because AD is designed to function without supervision, drivers will likely pay even less attention to the forward roadway than they do in manual driving. In line with this hypothesis, a previous driving simulator study reports a significant decrease in PRC from 74.5% in manual driving to 54% during AD, when the drivers were free to engage in non-driving related activities (Merat et al., 2012). Drivers who are looking away will need some time after the TOR to return their visual attention to the forward roadway. Research suggests that drivers on average direct their first glance toward the forward road within 2 s after the TOR in driving simulators (Gold et al., 2013; Vogelpohl et al., 2018; Zeeb et al., 2017), and within about 3 s on test track (Pipkorn et al., 2022). However, the time from the TOR to the first on-road glance may overestimate the level of visual attention to the road, since the driver may subsequently glance away. A more complete way of measuring drivers’ visual attention after the TOR is to compute the PRC over time in some specified time interval after the TOR. Merat et al. (2014) used this method, comparing drivers’ PRC 1 minute after TORs issued by AD systems, where drivers had to look to the forward roadway, using two different strategies. One strategy issued the TOR every 6 min and another issued the TOR if drivers looked off-road for 10 s or longer. For the TOR issued at fixed time intervals, drivers’ PRC was high (about 70%) at AD deactivation, 5 s after the TOR. The PRC then remained at similar levels until 15–20 s had passed. Although the TOR issued during long off-road glances showed the lowest PRC (58%) at AD deactivation, it increased to 80% when 15–20 s had passed. These results suggest that drivers with continual, long off-road glances during the TOR take longer to return to high levels of PRC following a TOR.

### Aim and Research Questions 

A detailed understanding of drivers’ visual attention before and after TORs in AD is required. Studies in real traffic are needed to support, and extend, previous findings made in virtual environments. It is also important to understand how drivers’ visual attention may change over time as they adapt to AD and get more practice in responding to TORs. For example, Zhang et al. (2019) found that as drivers gained more experience with TORs they deactivated AD more quickly. This and other possible learning effects may have an impact on drivers’ visual attention before and after TORs.

This study aims to understand drivers’ visual attention before and after a transition of control from AD to manual driving in real traffic. The following research questions were investigated:

(1) Where do drivers look during AD, and how long are the off-road glances?
(2) Where do drivers look after a TOR and when AD has been deactivated?
(3) What is the influence of repeated exposure to a TOR on drivers’ visual attention to the forward roadway before and after the TOR?

## METHOD

### Participants

Thirty participants, all employed at Volvo Cars in Gothenburg, were recruited for the study. One-third were females, and the mean age was 39.1 (SD = 10.5) years. All participants had driven at least 5000 km during the last year, and none of them worked as test drivers or were directly involved in the development of AD. Many of them had some familiarity with driving assistance systems: 83% used an Adaptive Cruise Control (ACC) and 50% used a Lane-Keeping Aid on a regular basis. This research complied with the tenets of the Declaration of Helsinki and was approved by the national ethical review board in Gothenburg, Dnr: 2019–01827. All participants signed a consent form prior to participation.

### Testing Environment and Equipment

#### The Public Road in Gothenburg

**Figure 1.**
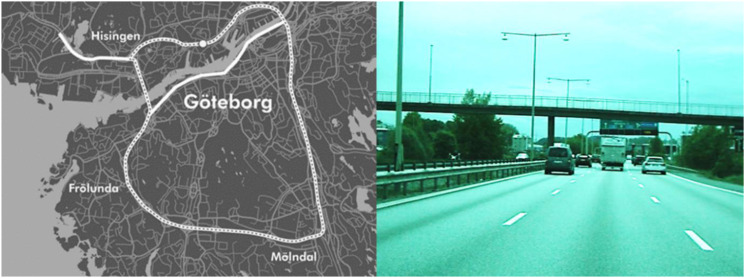
The public road in Gothenburg (the dashed line illustrates the stretch of road used in the study; left), with real traffic (right).

The study was conducted on a public road with real traffic in Gothenburg, Sweden (see Figure 1). One lap (dashed line in figure) was approximately 30 km long, and the posted speed was 70 or 80 kph. The road was mainly two or three lanes in each direction, with a median down the center.

#### The Wizard of Oz test vehicle

A *Wizard of Oz* (Green & Wei-Haas, 1985) setup was used in the study to simulate AD. The vehicle (a Volvo XC90) included a set of pedals and a steering wheel in the middle of the back seat which were visually obstructed from the test participants. Thus, AD was simulated by a *wizard* driving the vehicle from the back seat. The head and shoulders of the wizard were visible from the front seat, so the wizard’s presence was explained as a safety measure; the wizard would supervise the AD and only intervene if needed. The test vehicle was equipped with cameras that recorded video data of the participant (face and body) and the forward roadway.

**Figure 2.**
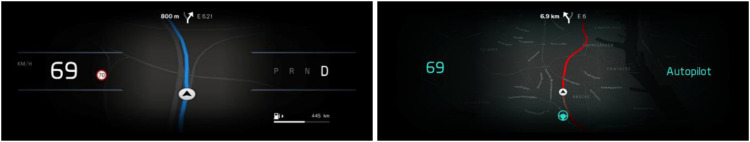
The instrument cluster display in manual mode (left), and when AD is active (right).

The AD was able to take full responsibility for the driving task without the need for supervision from the user. However, the user received a TOR when approaching the limits of the operational design domain (e.g., exiting highways). When AD was available for activation, the system notified the participant with an audio tone and a message in the instrument cluster (IC) directly in front of the driver which read, “Autopilot available.” The participant could then activate AD (i.e., cede control to the wizard) by pressing two buttons on the steering wheel for 0.6 s. The participants received feedback when AD was activated: a voice said, “Autopilot active” and the IC view was updated (Figure 2, right).

**Figure 3.**
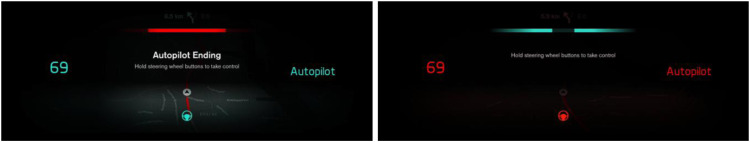
The instrument cluster view of the TOR (left) and the deactivation of AD (right), when the two steering wheel buttons are being pressed.

The TOR that informed the participants of the need to deactivate AD consisted of an audio tone and a message in the IC reading, “Autopilot ending” (Figure 3, left). When the TOR was issued, the participants had 6 s to deactivate AD (this time was illustrated in the IC with a shrinking red bar; see Figure 3, left). Deactivation was performed by pressing the two steering wheel buttons for 0.6 s (the same action performed for activation). The remaining time was illustrated in the IC with two turquoise bars (Figure 3, right) approaching each other and meeting when the deactivation was completed. When the AD was deactivated, the IC view changed to the manual driving mode (Figure 2, left) and a voice said: “Drive the car”.

### Study Procedure

The study consisted of a manual baseline drive and a test drive that combined manual driving and driving in AD mode. Prior to the study, all participants were asked to read an information sheet about driver responsibilities in manual driving and in AD. When in manual mode, the participants were required to obey traffic rules (e.g., keep to speed limits) and drive as they normally would. The participants were told not to use any driver assistance systems (e.g., ACC). When in AD, the participants were informed that they were not responsible for the driving task. They had the opportunity to bring items (e.g., magazine, notebook, or phone) of their choice to use while in AD mode. However, the participants were informed about the need to respond to potential TORs. Before the test drive started, the participants practiced activating and deactivating AD several times, both at standstill and during a short drive.

#### Baseline Drive

Manual baseline data was recorded while the participants drove manually (no assistance systems engaged) on the public road for two laps. The participants were unaccompanied.

#### Test Drive

**Figure 4.**
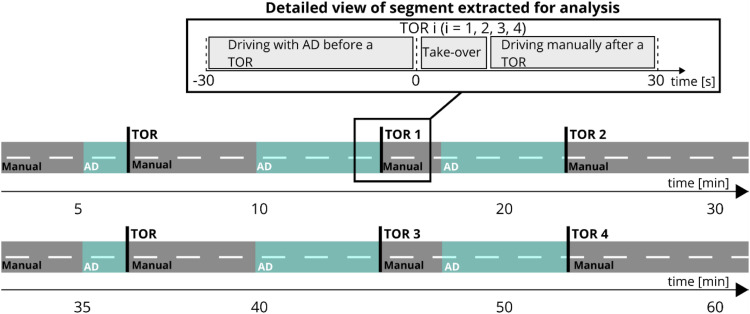
The participants completed two laps in total. Each lap included in the test drive started with manual driving and included two sections of road driven with AD. Each of the four AD drives ended with a TOR (TORs 1–4). For the analysis, 60 s segments (from 30 s before to 30 s after each TOR) were considered (see exploded view of segments). These segments included: driving with AD before the TOR, deactivation of AD (take-over) and manual driving after the TOR.

A test leader was present in the vehicle together with the test participant and the wizard. The test leader told the participants where to drive, but no other conversation took place. The participants drove two laps around the ring road (Figure 1) with a combination of manual and AD. The total duration of the test drive was about 60 mins (30 mins per lap). The test consisted of four AD drives that lasted four to 6 minutes and ended with a TOR (TORs 1–4 in Figure 4). At the start of each lap, the participants drove a 1-minute practice in AD to familiarize themselves with the system and the TOR.

### Data Processing and Coding

#### Selection of Video Segments

Video segments (recorded at 10 Hz) were selected for manual coding of the participants’ gaze direction during the baseline and test drives. Four video segments from 30 s before to 30 s after the TOR (see Figure 4) were extracted from each test drive (for a total of 120 video segments). Four segments were excluded due to deviations from the experimental protocol (one participant); another four segments were missing due to issues with observing the participant’s eyes for an extended time, resulting in a final sample of 112 video segments. In the baseline drives, four 30 s segments, corresponding to the same parts of road in the test drives, were extracted. Due to missing video (12 segments) and issues observing the participant’s eyes for more than 50% of the segment frames (nine segments), the final sample of manual baseline driving consisted of 99 video segments.

#### Coding Scheme for Gaze Direction to Areas of Interest

For the selected video segments, the participants’ gaze direction in each frame was manually coded, creating a time series of gaze directions with a time step of 0.1 s. The participants’ gaze direction in each frame was coded as a specific area of interest (AOI), according to a coding scheme inspired by the UDRIVE Annotation Codebook (Bärgman et al., 2017). The AOIs used in this study are On road, Side mirrors (including side windows), Rear mirror, Instrument cluster, Center stack (including glances to the mounted tablet), Peripheral interior, Peripheral exterior, Secondary task (for non-driving related task items), and Unknown. Any glance inside or outside the vehicle that was not towards any other AOI was coded as Peripheral interior (if inside) or Peripheral exterior (if outside). Glances toward handheld items (e.g., mobile phone, water bottle) were coded as Secondary task. Any glance that could not be determined because the participants’ eyes were not visible was coded as Unknown. If a majority of time steps within a segment were unknown these segments were excluded (as described in 2.4.1).

### Data Visualization and Analysis

#### The Distribution of Gaze Direction Toward Areas of Interest

To understand where drivers generally look before and after a TOR, the distribution of gaze direction towards each AOI (averaged over TORs 1–4) was plotted as a stacked histogram for each time step in the interval from 30 s before to 30 s after the TOR. Each 60 s segment was divided into four intervals (Intervals 1–4). The first interval corresponds to driving with AD (Interval 1). The second interval corresponds to the 6 seconds that participants were given to deactivate AD (Interval 2). The third and fourth intervals were defined by visual inspection of the stacked histogram (inspired by the work of Morando et al., 2016). The fourth represents the interval in which drivers return to visual attention levels similar to those in the manual baseline (Interval 4), and the transition between Intervals 2 and 4 is denoted as Interval 3. For comparison, a stacked histogram was plotted for a 30 s manual driving baseline and a 15 s interval (i.e., the middle portion of the 30 s interval) was marked in the same plot. The PRC was computed for each interval as the number of frames the gaze was directed to the forward roadway divided by the total number of frames within the same interval with known gaze directions. The median and standard deviation of PRC was computed and visualized on top of the stacked histograms. Interval 3 was not considered for statistical comparison since it represents a change in visual behavior from Interval 2 to Interval 4.

#### Duration of Off-road Glances

Off-road glance durations were computed for the 30 s of AD before the TOR and for a corresponding 30 s manual baseline. If these 30-s time intervals started or ended with an off-road glance, the complete glance duration was included (i.e., glances could be longer than 30 s). The off-road glance durations for automated and manual driving were plotted as cumulative frequency distributions expressed as percentages of all glances within each condition. In line with the work of Victor et al. (2018), the proportions of off-road glances that were longer than 2 s were computed for automated and manual driving, respectively.

#### The Influence of Repeated Exposure to Take-Over Requests on the Percentage Road Center

The PRCs in Intervals 1, 2, and 4 were summarized separately for each TOR with median and standard deviation. Further, to determine whether drivers’ visual attention to the forward roadway changes over time as drivers adapt to the take-over procedure, the PRCs in Intervals 1, 2, and 4 were compared across the four TORs.

#### Statistical Analysis

Statistical comparisons were performed using Friedman’s test (multiple non-normal distributions) and Wilcoxon signed-rank test (two non-normal distributions). The significance level (α) was set to 0.05/5 = 0.01 after correcting for multiple comparisons using Bonferroni correction.

## RESULTS

### Drivers’ Visual Attention Before and After a Take-Over Request

**Figure 5.**
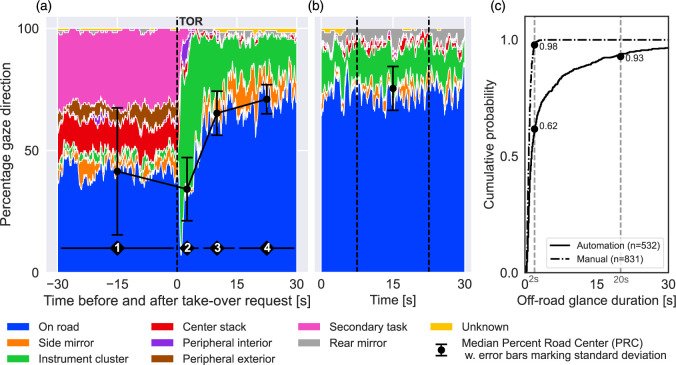
Panel a: Percentage of gaze direction to each AOI from 30 s before to 30 s after a TOR. The black diamonds mark four time intervals: (1) driving with AD, (2) the 6 s take-over process, (3) transitioning from interval 2 to 4, and (4) on-road glances stabilized during manual mode. Panel b: Percentage of gaze direction to AOI during a 30 s manual baseline (a 15 s baseline is marked with dashed lines). Panel c: The cumulative probability of off-road glance durations for AD and manual driving. The proportion off-road glances shorter than or equal to 2 and 20 s are indicated with black filled circles and numbers.

During AD (Figure 5a, Interval 1,−30 to 0 s), the percentage of gaze directed to the On road AOI (blue) was slightly lower (approx. 40%) than all the off-road AOIs combined. Among the off-road AOIs, Secondary task (pink) was the most common, followed by Center stack (red). In contrast, during manual driving (Figure 5b), the percentage of gaze directed to the On road AOI was markedly higher at approx. 75%, while the Instrument cluster (green) was the most common off-road AOI. The PRC was significantly lower during AD (Mdn = 41.2%) than during manual driving (Mdn = 75.4%, z = −3.975, *p* < .001, r =−.78). The off-road glance durations were also longer during AD: 62% of the off-road glances were shorter than or equal to 2 s in AD, compared to 98% in manual driving (see Figure 5c). In fact, during AD, 7% of the off-road glances were longer than 20 s, and the longest was about 5 min (i.e., almost the complete AD duration). In contrast, the longest observed off-road glance during manual driving was 3.6 s.

At the TOR (Figure 5a, at 0 s) the most common glance AOI was On road (37%) followed by Secondary task (34%) and Center stack (11%). In Interval 2 (0–6 s just after the TOR), glances towards the IC increased (Figure 5a). Drivers generally made repeated glances towards the IC. For 75% of the TORs, there were two or more glances to the IC in Interval 2; only 5% included just a single glance to the IC. The PRC was even lower in Interval 2 (Mdn = 34%, SD = 14%) than in Interval 1 (during AD). Importantly, 1 s after the TOR, the percentage of gaze direction toward the On road AOI was only 8%. Due to the difference in length between Intervals 1 and 2, no statistical comparison was performed.

In Interval 3 (6–15 s after the TOR), the percentage of gaze directed toward the IC decreased as the percentage gaze direction toward On road AOI increased (Figure 5a). In Interval 4 (Figure 5a, 15 to 30 s), drivers’ PRC returned to values similar to those of the manual baseline (Figure 5b). About 15 s after the TOR, the PRC seems to have stabilized (Figure 5a, Interval 4) with a median of 71.3%, which is not statistically significantly different from the PRC in manual baseline driving (Figure 5b, 15 s baseline, marked with dashed lines: Mdn = 71.4%, z = −2.67, *p* = .79, r = −.52).

### The Influence of Exposure to Take-Over Requests on the Drivers’ Visual Attention to the Forward Road

During AD (Interval 1), the median PRC was higher for the first exposure than for the following three (see Table 1). However, the differences in PRC were not statistically significant: x^2^(3) = 4.14, *p* = .25. The PRC during the transition from AD to manual (Interval 2) was similar for TORs 1–4 (see Table 1). In fact, the median PRC ranged from 35 to 37%, and the difference was not statistically significant: X^2^(3) = 2.94, *p* = .40. Finally, the PRC when the glances had returned to the road (Interval 4) did not differ statistically significantly across exposures under the Bonferroni correction: X^2^(3) = 7.84, *p* = .05.

**TABLE 1.** The PRC in Intervals 1, 2, and 4 (columns) across exposure to TORs (TORs 1–4, rows)

Exposure to TORs	PRC (%) Interval 1 (30 s) Mdn (SD)	PRC (%) Interval 2 (6 s) Mdn (SD)	PRC (%) Interval 4 (15 s) Mdn (SD)
TOR 1	56.5 (33.2), *n* = 27	36.7 (17.0), *n* = 27	71.3 (11.0), *n* = 26
TOR 2	25.2 (36.3), *n* = 27	35.0 (16.9), *n* = 27	72.7 (11.60), *n* = 26
TOR 3	27.6 (31.1), *n* = 27	35.0 (15.3), *n* = 27	69.3 (9.4), *n* = 26
TOR 4	33.6 (37.7), *n* = 27	36.7 (16.4), *n* = 27	73.0 (9.6), *n* = 26

## DISCUSSION

### Drivers’ Visual Attention Before and After a Take-Over Request

Using data collected on public roads, the present study provides novel findings regarding drivers’ visual attention during AD and the transition of control to manual driving. The study also confirms previous findings from driving simulators: drivers pay less attention to the forward road when driving with AD than during assisted or manual driving. Instead of looking towards the forward road, many of the drivers mainly used the time in AD to look towards non-driving related task items (e.g., mobile phone, or tablet mounted on top of the center stack). In fact, PRC was even lower in real traffic (41%) than in a previous driving simulator study (54%; Jamson et al., 2013). Moreover, the off-road glance durations were markedly longer with AD than in previous studies with ACC and assisted driving features. The present study observed off-road glances up to 5 min long, corresponding to almost the full AD duration, whereas Victor et al. (2018) observed maximum off-road glance times of 40 s for an assisted driving system on a test track. Note, though, that they had instructed the drivers to supervise the driving, whereas in the present study the drivers were allowed to look away from the road as much as they wanted during AD. The present study also observed a greater percentage of off-road glances longer than 2 s (38%) than reported in previous studies: 14.5% in Victor et al. (2018) for an assisted driving system on a test track, and 4% in naturalistic driving with an ACC and lane-keeping aid (Morando et al., 2019). During AD, drivers are allowed to engage in non-driving related tasks; therefore, they may be inattentive. It is important that AD be designed to always issue a timely TOR when drivers are expected to resume manual driving.

Importantly, the present study observed a noticeable lack of visual attention to the forward roadway just after the TOR. More specifically, only 8% of gaze was directed towards the forward roadway 1 s after the TOR. This finding suggests that issuing a TOR may, in fact, have an unexpected, opposite effect directly after the TOR. Instead of triggering drivers to look forward, the TOR may cause drivers to look toward the IC to understand the message in the display. Importantly, these off-road glances just after the TOR increase the risk that drivers will miss important environmental cues needed for safe manual driving and responding to events (e.g., a stationary vehicle ahead). Finally, based on the PRC results, drivers generally start to be as attentive as in normal manual driving 15 s after the TOR.

Our findings suggest that previous research focusing on drivers’ take-over times may overestimate their ability to safely drive manually after AD. In fact, the average reported take-over time, based on 129 studies, is 2.7 s. In addition, the most commonly reported amount of time available for drivers to resume manual control in response to a TOR (the take-over time budget) is 7 s (McDonald et al., 2019; Zhang et al., 2019). According to our findings, at both 2.7 and 7 s after the TOR some drivers may still be looking more off-road than they would be in manual driving. They might therefore miss safety-critical events, despite being fully responsible for driving since AD has been deactivated. In addition, previous studies that only consider a single visual response time (the time needed for drivers to look towards the forward road in response to a TOR) may also overestimate drivers’ ability to perform safe manual driving. While previous studies report that, on average, drivers first glance back to the forward roadway within 2–3 s after the TOR, our findings suggest that drivers may have several repeated off-road glances after the initial on-road glance. Further, unlike Merat et al. (2014), we did not observe high PRC levels 5–10 s after the TOR. However, drivers in that study were directed to look at the forward road even during AD, while the drivers in our study could engage in visual non-driving related tasks. Ending a non-driving related task before looking at the forward roadway results in a time delay that does not exist for those already looking forward. Therefore, it is not surprising that our findings are more in line with the PRC observed in Merat et al.’s study, when the TOR was issued during a long off-road glance. That is, in their study the PRC had reached over 70% 15–20 s after the TOR and in the present study the PRC had reached 70% 15–30 s after the TOR.

In sum, our findings suggest that a TOR triggers off-road glances, and it can take 15–30 s before drivers devote the same level of visual attention to the forward roadway that they did during manual driving. When TORs include visual information in the IC, it is important that their design ensures that drivers have enough time to receive this information before they are expected to respond to events in traffic. Alternatively, TORs could be designed to encourage drivers to look forward, rather than away from the forward road: one possibility is a head-up display (HUD) that would not require drivers to look at the IC. However, it still cannot be assumed that the driver is sufficiently aware of the road environment (i.e., driver may focus on the message and not on the forward road), even though the transition time is likely shorter from the HUD than from the IC.

### The Influence of Exposure to Take-Over Requests on the Drivers’ Visual Attention to the Forward Road

The present study did not observe significant differences in driver attention toward the forward roadway when drivers experienced AD and TORs four times during the complete drive (i.e., repeated exposure). The median PRC was slightly higher during AD leading up to the first TOR (TOR 1) compared to TORs 2–4. However, the individual variation across drivers was large, as is evident in Interval 1 in Table 1. During AD, some drivers chose to look away for most of the time while some preferred to look mainly at the road. This difference in visual behavior can be explained by a difference in individuals’ willingness to engage in non-driving related tasks, which in turn may be influenced by factors such as trust in automation (Hergeth et al., 2016) and motion sickness (Dam & Jeon, 2021).

Further, repeated exposure to TORs did not appear to influence the PRC, either during the transition of control or during the manual driving after the transition. However, our data suggest a tendency for a learning effect to influence drivers’ visual attention during AD before the first TOR (first exposure compared to the remaining three in Interval 1), but not after the TOR. However, since the present study only exposed the participants to AD over one day, the influence of repeated exposure to AD over several days remains unknown. It is possible that, over time, the attention levels observed in our study would become even lower in a similar study lasting many days. A driving simulator study by Metz et al. (2021) supports this possibility; they found that with increasing use (six experimental sessions on different days) the participants increased their trust in and acceptance of the system, reducing their visual attention towards the forward roadway. On the other hand, repeated exposure could cause drivers to learn to associate the TOR’s audio tone with the need to deactivate AD and consequently, to look less towards the IC (and more towards the road) in response to a TOR. The result over time of these combined effects—looking less toward the road due to increased trust and looking more towards the road due to familiarity with the TOR procedure—remains unclear. Thus, a longitudinal study (over days) using the same type of Human-machine interface as in our study, in naturalistic settings, is needed to clarify the combined effect of learning the TOR procedure and increasing trust in AD.

### Limitations and Future Work

The findings presented in this paper are based on a Wizard of Oz setup, which provides a higher degree of realism than the driving simulator and test track studies in previous literature. However, the results may have been influenced by the presence of a test leader and a wizard driver in the vehicle. In particular, the long off-road glances may be partly due to participants’ feeling of increased safety because of the wizard driver’s presence (explained as a safety precaution). As a further limitation, the participants may not be representative of an international population (in part because they are Volvo employees, even though were not involved in work related to AD product development). To explore how the present results generalize to driving in real traffic across a wider population, a future study should investigate drivers’ visual attention using naturalistic driving data. Finally, the present study mainly focused on visual attention in terms of distributions of gaze towards certain areas of interest—in manual driving and in AD (before and after TORs). Future studies with advanced eye tracking systems which can record metrics such as fixation durations and frequencies and blink patterns would allow more detailed analyses of drivers’ visual attention.

## CONCLUSION

AD allows drivers to disengage from the driving task since there is no need for supervision of the driving when AD is activated. While some drivers will mainly look on-road, others may use this opportunity to engage in non-driving related activities and therefore look considerably less on-road than when driving manually. Some drivers may look off-road almost the entire time the AD is on. Thus, it cannot be assumed that drivers are sufficiently aware of the driving environment during AD to control the vehicle appropriately if a critical situation arises suddenly. Events that require drivers' detection and response without a vehicle notification could result in severe crashes when drivers are engaged in non-driving related activities since their attention is directed elsewhere. Thus, it is essential that AD takes full responsibility for the complete driving task when activated and always issues a take-over request when a transition to manual control is necessary. Further, a take-over request design that includes visual information in a display (e.g., instrument cluster) immediately attract drivers’ visual attention away from the forward road. Thus, drivers may miss critical visual cues from the forward roadway that may be important for safe manual driving and responding to events. In fact, in our study, it took 15–30 s after the take-over request before the drivers' visual attention to the forward roadway returned to the same level as in manual driving. Furthermore, these findings were stable across exposures (drivers received several take-over requests during the same 1 h drive). However, more extensive longitudinal studies in a naturalistic setting are needed to understand how drivers' attention may change as they familiarize themselves with the take-over request.

## KEY POINTS

- In automated driving (AD), drivers reduce their visual attention to the forward road and increase the duration of single off-road glances compared to manual driving.
- AD provides the opportunity for drivers to look away from the road (e.g., at a mobile phone or a tablet) and many drivers do not hesitate to do so.
- In response to take-over requests, drivers look toward the instrument cluster. Thus, the request may trigger off-road glances and hinder drivers from understanding important visual cues from the forward roadway that are important for safe manual driving after AD.
- After a take-over request, it takes at least 15 s for drivers to show visual attention to the road at the levels observed in manual driving.
- Short-term repeated exposure to take-over requests does not seem to influence drivers’ visual attention to the forward road, either before or after a transition of control from AD to manual driving.
